# Genetic Analyses and Genome-Wide Association Studies on Pathogen Resistance of *Bos taurus* and *Bos indicus* Cattle Breeds in Cameroon

**DOI:** 10.3390/genes12070976

**Published:** 2021-06-26

**Authors:** Babette Abanda, Markus Schmid, Archile Paguem, Hanna Iffland, Siegfried Preuß, Alfons Renz, Albert Eisenbarth

**Affiliations:** 1 Department of Comparative Zoology, Institute of Evolution and Ecology, University of Tübingen, 72076 Tübingen, Germany; achillepaguem@yahoo.fr (A.P.); alfons.renz@uni-tuebingen.de (A.R.); albert.eisenbarth@bnitm.de (A.E.); 2 Institute of Animal Science, University of Hohenheim, 70599 Stuttgart, Germany; markus_schmid@uni-hohenheim.de (M.S.); hanna.iffland@uni-hohenheim.de (H.I.); Siegfried.Preuss@uni-hohenheim.de (S.P.); 3 Department of Biological Sciences, University of Ngaoundéré, Ngaoundéré, Cameroon

**Keywords:** SNP chip, heritability, case-control, parasitic disease, cattle

## Abstract

Autochthonous taurine and later introduced zebu cattle from Cameroon differ considerably in their resistance to endemic pathogens with little to no reports of the underlying genetic make-up. Breed history and habitat variations are reported to contribute significantly to this diversity worldwide, presumably in Cameroon as well, where locations diverge in climate, pasture, and prevalence of infectious agents. In order to investigate the genetic background, the genotypes of 685 individuals of different Cameroonian breeds were analysed by using the BovineSNP50v3 BeadChip. The variance components including heritability were estimated and genome-wide association studies (GWAS) were performed. Phenotypes were obtained by parasitological screening and categorised in Tick-borne pathogens (TBP), gastrointestinal nematodes (GIN), and onchocercosis (ONC). Estimated heritabilities were low for GIN and TBP (0.079 (se = 0.084) and 0.109 (se = 0.103) respectively) and moderate for ONC (0.216 (se = 0.094)). Further than revealing the quantitative nature of the traits, GWAS identified putative trait-associated genomic regions on five chromosomes, including the chromosomes 11 and 18 for GIN, 20 and 24 for TBP, and 12 for ONC. The results imply that breeding for resistant animals in the cattle population from Northern Cameroon might be possible for the studied pathogens; however, further research in this field using larger datasets will be required to improve the resistance towards pathogen infections, propose candidate genes or to infer biological pathways, as well as the genetic structures of African multi-breed populations.

## 1. Introduction

In cattle breeds, the genetic makeup has been shown to create different phenotypes related to their ability to sustain environmental pressure, including pathogens. Particularly in developing countries, many of the endemic pathogens considerably affect the cattle livestock industry [1], for instance endoparasitic infections caused by a single or mixture of gastrointestinal nematodes (GIN) such as *Toxocara*, Strongyles, *Strongyloides*, and *Trichuris* [2,3]. These infections lead to losses in meat and milk production systems. Additionally, female infertility and rapid death in younger animals may occur after infection without warning signs [4]. The resistance to tick bites via immunogenic reactivity against compounds is in the tick salivary, and thus the likelihood to acquire tick transmissible microorganisms is reported to vary among different breeds of cattle [5,6]. This combination of ticks and transmissible diseases (tick-borne pathogens: TBP) is known to be a substantial drawback for the improvement of the livestock sector in sub-Saharan Africa. As an example, in many regions of Africa, the tick species *Rhipicephalus* (*Boophilus) microplus* is progressively invading new areas with published reports of displacing established populations of closely-related species [7].

Cameroon is a country situated in Central Africa and home to both autochthonous taurine (*Bos taurus)* and zebu (*Bos indicus)* cattle breeds, as well as to all agents of the above-mentioned infectious diseases. The respective distribution of those pathogens is thereby dependent on the climatic zone, habitat preference and vector abundance. The whole genomes of individuals of the investigated breeds have been recently published [8]. The latter study revealed hints of considerable breed admixture, possibly due to uncontrolled mating. To assess the possible exploitation of economically valuable phenotypic traits, heritability studies have been used all over the world evaluating which of the related traits may be targets for breeding improvement [9,10,11]. In Cameroon, heritability studies on cattle resistance to parasites have been done for ticks [12,13], but no clear genetic effect could be demonstrated. In the human host, substantial inter-individual variations of infection burden with the tissue-dwelling filarial nematode *Onchocerca volvulus* were not explainable by exposure to parasite transmission, suggesting a substantial genetic predisposition [14]. In the cattle host, exposed but non-*Onchocerca* *ochengi* infected individuals have been described as putative immune [15], with no gene association study yet carried out. Although most species of the bovine *Onchocerca* (ONC) parasites have been reported non- or low-pathogenic [16], their importance mainly being a naturally occurring model for the closely-related human parasite. Traits associated with disease resistance have already been found to be heritable, with identified regions mapped by genome-wide association studies (GWAS), even if at times with low estimates [9,17].

The present study aims to investigate the genetic background of the resistance against pathogens by using the infection status of individuals with regard to vector-borne (TBP and ONC) and oral-faecal-transmitted (GIN) pathogens in cattle breeds from Cameroon.

## 2. Materials and Methods

### 2.1. Sample Collection

**Figure 1:**
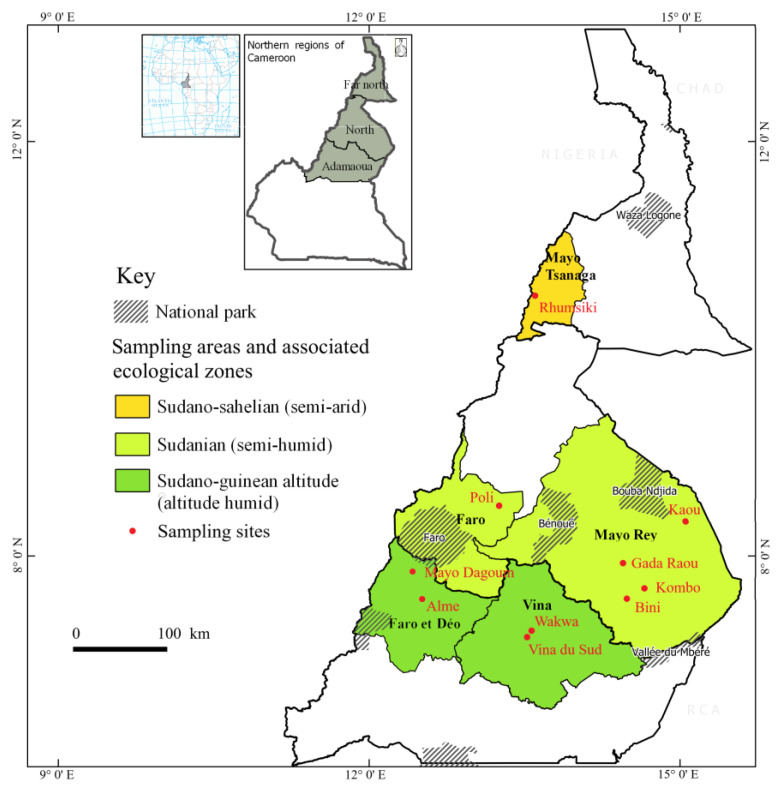
Sampling. The Vina and Faro et Deo sites are located in the Adamawa region, the Faro and the Mayo-Rey in the North region, and the Mayo Tsanaga in the Far North region. Ecological zones and climatic conditions of each of the sampling zones are shown. The coloured zones represent the sampling areas, the zones with stripes the national parks, and the red dots the sampling sites. Image adapted from [22].

As large datasets are indispensable for reliable results in genomic studies [18], a pooled multi-breed dataset was examined. A total of 1260 cattle were examined, of which a subset of 719 (57%) was selected for genotyping analyses. Thirty-four samples did not meet the quality control criteria and were excluded for the analysis. The sample set originated from five districts in North Cameroon located in the provincial regions Adamawa, North and Far North where approximately 83% of the national cattle population is located [19], and consisted of 719 individuals from the *Bos indicus* breeds Fulani (*n* = 100) and Gudali (*n* = 372), as well as the autochthonous *Bos taurus* breeds Kapsiki (*n* = 137) and Namchi (*n* = 110). Additional information is available from [20,21]. The study data collection took place in both dry and rainy seasons of the year 2014. The sampling sites spanned across three different bioclimatic zones: the sub-humid Guinea savannah biotope of the Adamawa highlands, the semi-arid Sudan savannah in the North region, and the arid Sahel region in the region Far North (Figure 1). The regions were subdivided into five sites according to the availability of samples and the willingness of the owner to participate in the study. The number of individuals after/before quality control for each district, respectively, were: Vina (*n* = 123/125) and Faro et Deo (*n* = 105/128) in the Adamawa region; Faro (*n* = 106/110) and Mayo-Rey (*n* = 215/219) in the North region, and Mayo Tsanaga (*n* = 136/137) in the Far North region. The quality control ensured that individuals without reliable phenotypic records or more than 10% missing genotypes were discarded. The sample collections have been carried out with the consent of the regional state representatives and traditional authorities from each of the sampling areas. Furthermore, oral consent was given by the cattle owners, herdsmen (who also helped in restraining the animals), and with the participation and approval of the National Institute of Agricultural Research for Development (IRAD) in Cameroon, which is the country’s government institution for animal health and livestock husbandry improvement.

### 2.2. Pathogen Identification and Phenotype Classification

DNA was extracted from blood, as previously reported [20]. Phenotypic information was obtained using molecular detection for TBP, faeces treated by flotation technique for GIN, and small skin biopsies from the inguinal region for ONC. Details of the TBP identification by molecular tool, distribution and biodiversity is given in Abanda et al. [20]. Isolated eggs of GIN were identified by the McMaster technique [23], *Onchocerca* spp. were investigated by intradermal nodule palpation [24] and isolation and identification of microfilariae of *O. ochengi*, *O. gutturosa* and *O. armillata* according to Wahl et al. [16] for ONC. All phenotypes were binary coded as 1 (infected) and 0 (not infected).

### 2.3. Genotyping

Genotyping was conducted using the Illumina BovineSNP50v3 BeadChip (Illumina, San Diego, CA, USA). After quality control, all annotated autosomal single nucleotide polymorphisms (SNPs) with a minor allele frequency (MAF) ≥ 0.05, no significant deviation from Hardy-Weinberg-equilibrium (*p* < 0.001), and segregating in all of the breeds were favoured for downstream analyses. The final dataset contained the phenotypes of 608 to 685 animals depending on the trait and their genotype status of 35,195 SNPs (Supplementary Table S1).

### 2.4. Statistical Analysis

The statistical analysis consisted of two major sections, the estimation of variance components, and the estimation of marker effects in order to infer marker-trait-association. Both were conducted using the software GCTA (version 1.91.7beta) [25]. Initially, all available fixed effects (sex, age, season_year, breed_site) were tested for significance (*p* < 0.05) to determine the effects to be included in the evaluation model. Since not all breeds were present at all sites, and samples were not taken in all seasons in each year, combined effects (breed_site and season_year) were considered. The heritabilities were calculated based on the estimated variance components using standard notations. For the variance component analyses, the following model was applied:

(1) y=Xb+g+e

where vector y contains the phenotypes of the individuals, b denotes the fixed effect breed_site (additionally age for the trait ONC), and X is the corresponding design matrix. g is the random genetic animal effect, with $g\;\sim \;N\left(0,G\sigma_{g}^{2}\right)$ and G being the genomic relationship matrix. The vector e includes the residuals, with $e\sim N\left(0,I\sigma_{e}^{2}\right),\;$ where *I* is the identity matrix. As the traits were binary coded, heritabilities were calculated on the observed, as well as on the unobserved liability scale (λ), and given assumed population prevalences of 0.8, 0.6 and 0.5 for the traits GIN, ONC, and TBP, respectively. Thereby, *λ* is obtained by linear transformation of the observed binary coded phenotype to an unobserved continuous scale. In order to estimate the level of association between the traits and the significant SNPs in GWAS, model 1 was extended towards:

(2) y=Xb+Wu+g+e

Thereby, u denotes the fixed effect of the SNP to be tested and W the design matrix containing the number of 1-alleles. A leave-one-chromosome-out (loco) approach was applied to avoid a loss in mapping power by double-fitting the tested SNP. Due to the limitation of the sample size and the associated produced dataset, a Bonferroni corrected genome-wide significance level was too stringent to be applied, hence SNPs with *p*-values smaller than the threshold of $p_{nominal}=5\times 10^{-5}$ were assumed to show nominal significant trait association. The genome-wide significance level was defined as $p_{genome-wide}=\frac{0.05}{n},$ where *n* denotes the number of SNPs.

## 3. Results

Parasitological examinations representing phenotypic data are reported in Table 1 including the cattle breed, the pathogen considered, and the respective distribution according to the site of sampling for TBP, ONC, and GIN. The fixed effect breed_site had a significant impact on all traits whereas the age-effect was solely significant for ONC.

The estimated variance components, as well as heritabilities based on the observed and the liability scale are given in Table 2. Phenotypic variances with low standard errors were estimated ranging from 0.063 to 0.181. A high or low observed pathogen prevalence in the population came along with considerably smaller estimates. The greatest phenotypic variance was by far observed for ONC, for which the prevalence was rather close to an intermediate value. Analogous results could be observed for the estimated additive genetic variance resulting in low heritabilities on the observed scale for all traits but ONC ($h_{obs.}^{2}=0.216$). The standard errors were generally large for the estimates of the additive genetic variance and the heritabilities. The heritability estimates were substantially higher when the estimation was based on the liability scale and resulted in moderate to high heritabilities for all investigated traits. However, the standard errors were large and even exceeded the estimates in the case of GIN.

**Figure 2:**
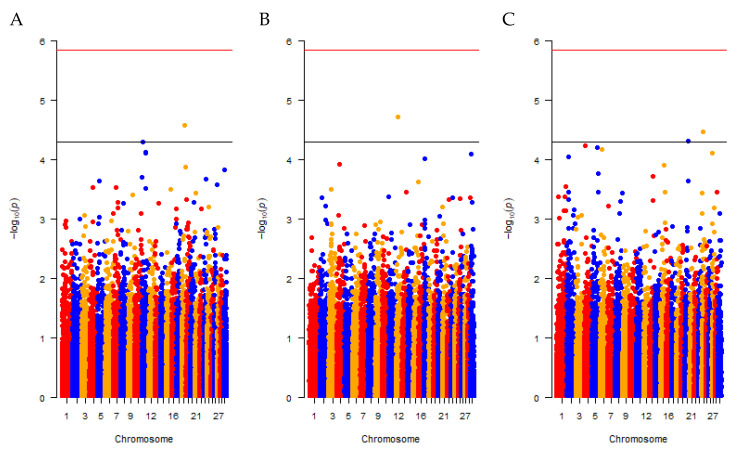
Manhattan plots for the investigated traits. The −log_10_-*p*-values of the SNP effects and their chromosomal positions are shown for the traits gastrointestinal nematodes (**A**), onchocercosis (**B**) and tick-borne pathogens (**C**). The horizontal bottom black line corresponds to a nominal significance level of $p_{nominal}=5\times 10^{-5}$, the top red line to the genome-wide significance level.

Figure 2 displays the GWAS results for each of the studied traits. For all analysed traits, the majority of SNP effects were small; however, several regions harbouring SNPs with obviously larger effects were distributed across almost all chromosomes. Two SNPs on chromosome 11 and 18 exceeded the nominal significance threshold indicating putative trait-associated genomic regions for GIN (Figure 2A), and one on chromosome 12 for ONC (Figure 2B). For TBP (Figure 2C), one significant SNP was found on chromosome 20 and another one on chromosome 24. None of the reported outlying SNPs reported from the traits reached the genome-wide level of significance.

## 4. Discussion

As in many parts of the world, the distribution and phenotypic adaptation of cattle in Cameroon has been strongly influenced by herd migration history, climate, wildlife abundance, vector and pathogen occurrence, and food and water accessibility [26,27,28,29]. Our results showed that the fixed effect breed_site had a significant association with the acquisition of all investigated pathogens. This was expected because the studied sites’ climate differed widely from humid to sub-humid and arid conditions [29,30]. Further, genetic studies observed differences between *Bos taurus* and *Bos indicus* breeds and have shown that genetic variation plays a significant role in terms of an animal’s resistance towards parasites as reviewed by Mapholi et al. [31,32]. Given the present dataset, the effect of the breed and the site could not be distinguished due to the heterogeneous distribution of all breeds not present at every site. Consequently, breeds were not separately analysed as large datasets are needed to obtain reliable results in genomic studies, and pooling data being a suitable approach to increase the power in such studies and to strengthen the results [18]. The observed significance of the age effect in the acquisition of ONC can be explained by the cumulative character of the long-living parasite population over time. To prove that statement in the present study, we grouped all individuals according to their age (<3 years, >3 years) and calculated the prevalence of infection within these groups. Thereby, 73% of the individuals that were older than 3 years were infected, whereas younger individuals were less affected (46%). This evidence has been previously demonstrated in Gudali cattle from the Adamawa region in Cameroon [33]. Indeed, susceptible individuals grouped into early and late susceptible had increasing nodules and microfilarial loads with significant differences in the prepatency period [24,33] variable from male to female, but with no visible gender effect in our dataset. 

The variance component estimation uncovered the susceptibility of individuals towards infections with the studied pathogens to be to some extent controlled by genetics. As expected for binary coded traits, a high or low observed pathogen prevalence in the population came along with considerably smaller variance component estimates [34]. Compared with the other traits, obviously larger variance component estimates could be presented for ONC, for which the pathogen prevalence was rather close to an intermediate observed prevalence. In agreement with [35], all heritabilities were substantially higher when the estimation was based on the liability scale since these also capture parts of non-additive genetic variance, particularly for traits where the prevalence is close to zero or unity [34]. The standard errors were generally large for the estimates of the additive genetic variance and the heritabilities, mainly since the number of individuals was limited and the multi-breed data structure was complex. The results imply that breeding might be possible to improve pathogen resistance in Cameroonian cattle for all three traits investigated here. However, as the variance components are population-specific parameters and are additionally influenced by the prevalence within a population [36], further studies using large representative datasets of the breeds are required to accurately determine the extent of the genetic variation or the response to selection within certain breeds. The visualised GWAS results (Figure 2) are characterised by numerous small and a few larger SNP effects across all chromosomes implying a quantitative trait nature for each of the traits. Several putatively trait-associated regions were identified for all of the traits. The region located on chromosome 24 having an impact on TBP has not been reported as an association signal elsewhere [37]. Generally, for all traits investigated, a relatively small number of neighbouring SNPs seems to be in strong linkage disequilibrium (LD) with the significant SNPs, which in some cases led to single significant SNPs instead of clear peaks, i.e., clear association signals (see Figure 2). This might be attributed to the multi-breed dataset, for which a large effective population size can be assumed and hence LD decays fast [38,39]. Additionally, due to data filtering, the number of SNPs can be decreased in chromosomal regions where an increased number of SNPs do not segregate in all of the breeds. As a Bonferroni corrected genome-wide significance level is too stringent, especially when the tests are not independent, like in the present study, an additional nominal significance level was applied to detect regions with weak associations. Due to the limited statistical power resulting in a lack of genome-wide significant SNPs, post-GWAS analyses were not conducted. In order to actually map and discuss quantitative trait loci (QTL), larger sample sizes are required. Hence no further discussion of the association signals was intended here.

A plethora of constantly updated reports [17,40,41] of SNPs associated with TBP and GIN, as well as the heritability estimates between low to medium values in the present study (see Table 2) confirm the importance of genomics for livestock improvement. Even though this study has discovered findings of such potential, they should be interpreted with caution due to the large standard errors of the estimates. In future investigations, large-scale studies or meta-analyses might give a better insight into the architecture of the traits in future GWAS. Special attention should be payed to LD structures in multi-breed populations as investigated here because LD consistency is compromised across populations [42]. In summary, the results imply that breeding for resistant animals in the cattle population from Northern Cameroon might be possible for the studied pathogens. Nevertheless, the findings suggest that further research in this field using larger datasets will be worthwhile to improve the resistance towards pathogen infections and to infer the genetic structures of African multi-breed populations. The latter is of great importance because uncontrolled admixture is predominant in the breeds investigated [8]. In fact, individual herd performances of local adaptation and resistance to pathogens can greatly vary based on the management system and the level of introgressed genes. It is therefore recommended to conduct interdisciplinary studies where genetic parameters are supplemented with environmental factors, information of the husbandry system, and molecular-diagnostic analyses for local pathogens diversity and exposure, together with productivity determinants.

## 5. Conclusions

The present study is the first in-depth investigation of the genetic makeup towards pathogen resistance of the cattle population in Cameroon. For all the studied groups of endemic pathogens genetic determinants were found, but due to the combination of high population variability and the limited sample size, a clear association with the involved genes was not possible. Future genetic association studies should consider these circumstances already in their planning phase. 

## Figures and Tables

**Table 1:** Characteristics of phenotyped and genotyped dataset used in the analysis, including the sampling site and parasite burden.

				GIN ^1^				ONC ^2^		TBP ^3^		
Site	Cattle Breed	*n*	Species	+	NA	-	+	NA	-	+	NA	-
Mayo Tsanaga	Kapsiki	136	*B. taurus*	118	8	10	110	0	26	134	0	2
Faro	Namchi	106	*B. taurus*	80	0	26	52	1	53	95	0	11
Mayo Rey	Fulani	26	*B. indicus*	25	0	1	24	0	2	21	0	5
	Gudali	189	*B. indicus*	188	0	1	149	2	38	169	0	20
Vina	Gudali	123	*B. indicus*	117	0	6	75	7	41	116	0	7
Faro et Deo	Fulani	68	*B. indicus*	47	2	19	1	66	1	67	0	1
	Gudali	37	*B. indicus*	26	0	11	11	1	25	36	0	1
TOTAL		242	*B. taurus*	198	8	36	162	1	79	229	0	13
		443	*B. indicus*	403	2	38	260	76	107	409	0	34
		685		601	10	74	422	77	186	638	0	47

^1^ gastrointestinal nematodes, identified by McMaster faeces flotation: *Toxocara* spp., Strongyle spp., *Strongyloides* spp., *Trichuris* spp. ^2^ onchocercosis, identified by skin biopsies and/or palpation *: *Onchocerca ochengi* *, *O. gutturosa*, *O. dukei*, *O. armillata*. ^3^ tick-borne pathogens, identified by PCR: *Anaplasma* spp., *Borrelia* spp., *Rickettsia* spp., *Theileria* spp. +: tested positive; NA: test result not available; -: tested negative; * indicates palpation. All species marked with asterisk were detected by palpation. The rest only by skin biopsies.

**Table 2:** Population specific parameters of the investigated traits. The estimated phenotypic (V_P_) and additive genetic (V_A_) variance, the heritability estimated on the observed ($h_{obs.}^{2}$) and liability scale ($h_{liab.}^{2}$) as well as their standard errors (in parentheses) are shown. The number of evaluated individuals (*n*) and the observed prevalence in the investigated population are given.

Trait ^1^	*n*	Prevalence	$V_{P}\;(\mathrm{SE})$	$V_{A}\;(\mathrm{SE})$	$h_{obs.}^{2}\;(\mathrm{SE})$	$h_{liab.}^{2}\;(\mathrm{SE})$
GIN	675	0.890	0.087 (0.005)	0.006 (0.007)	0.079 (0.084)	0.265 (0.281)
ONC	608	0.694	0.181 (0.011)	0.039 (0.017)	0.216 (0.094)	0.393 (0.170)
TBP	685	0.931	0.063 (0.003)	0.007 (0.006)	0.109 (0.103)	0.666 (0.631)

^1^ gastrointestinal nematodes (GIN), onchocercosis (ONC), tick-borne pathogens (TBP).

## Data Availability

Data is contained within the article. For further enquiries contact the corresponding author here guimbangabanda@yahoo.fr.

## Supplementary Materials

The following are available online at https://www.mdpi.com/article/10.3390/genes12070976/s1, Table S1: SNP profile of the analysed cattle generated by the Illumina Bovine SNP50v3 chip.
